# Diphtheria and Anti-Toxin

**Published:** 1896-01

**Authors:** 


### DIPHTHERIA AND ANTI-TOXIN.

The Homœophatic Society of Chicago held its regular monthly meeting on the evening of January 9th, in the Great Northern Hotel. Except the World's Fair Congress, it was the largest gathering of homœopathic physicians we have ever seen in Chicago. More than two hundred were present, and many were unable to get into the hall. The large crowd came out to hear the anti-toxin treatment of diphtheria discussed.

Dr. R. N. Tooker read a paper entitled "The Status of Diphtheria Anti-toxin at Home and Abroad." The Society listened to him with marked attention for a full hour. He was followed by a paper, or lecture, by Dr. J. A. Tomhagen, entitled "The Homœopathic Treatment of Diphtheria." He occupied a half-hour of the time of the Society. The first essayist had not a single word to say in favor of anti-toxin, but said much against it. He occupied a large part of the hour in showing up the misleading statements of the health department of Chicago. He claimed that the death-rate in Chicago from diphtheria during the last few weeks of 1895 was 44 per cent. instead of six per cent., as recently stated by the health commissioner. The essayist also said that during the year 1895 there were nearly 1,800 deaths in Chicago from diphtheria—500 more than ever occurred during any other year. He also claimed that the death-rate was even greater since the use of anti-toxin than before. He quoted statistics to show that the best average recent mortality rate which the old school could show was 22 per cent. He said he had addressed a circular letter, with a blank, to all the physicians of the homœopathic school in Chicago, and forty-five replied that, during the twelve months just preceding, they had treated in the aggregate 315 genuine cases with a death-rate of seven and three-tenths per cent. He believed that these statistics which he obtained thus were reliable, and correctly represented the average mortality rate in diphtheria under homœopathic treatment. According to these figures, if all the cases of diphtheria in Chicago, during the year 1895, had been treated homœopathically a thousand lives would have been saved. The action of the Chicago Board of Health in persisting in thrusting anti-toxin upon the profession was condemned in strong language. In this he evidently had the sympathy of the audience. The folly of the city of Chicago in spending from $300 to $500 a day in carrying out the anti-toxin "fad" was emphasized.

A committee was appointed to draw up resolutions protesting against the presumption of the Health Commissioner in dictating to the profession of Chicago how diphtheria should be treated. The present Commissioner of Health of Chicago is not a physician at all, but a very able ward politician who bases his greatest qualification to occupy the position he holds upon his "horse sense." The fear was expressed that his horse sense was no better than his "horse serum." A protest was made against filling the position of Health Commissioner of Chicago with an individual who has no knowledge of medicine. A number of physicians openly stated that they would not report their cases of diphtheria to the Health Department, as the regulations require, because their patrons would be urged at once to use anti-toxin.

In his circular of inquiry to the homœopathic profession of Chicago, Dr. Tooker requested the names of the remedies found most useful in diphtheria. Kali-bichromicum was mentioned 28 times and Mercurius-cyanide 27 times. A number of other remedies less frequently.

Dr. Tomhagen made a strong plea for a single dose of the indicated remedy in a high potency. He condemned all local applications, not only in diphtheria but in all other diseases. He spoke of the value of Lycopodium and Lachesis in diphtheria, the former being indicated when the disease began on the right side and was worse at 4 P. M.; the latter being demanded when the disease began on the left and extended to the right side. Other indications of Lachesis were marked aggravation after sleep and more pain in the skin of the neck than in the throat.

On account of the lateness of the hour the discussion was not completed. Anti-toxin received but the faintest praise from one or two speakers.

